# Novel Treatment Strategies for Glioblastoma

**DOI:** 10.3390/cancers12102883

**Published:** 2020-10-08

**Authors:** Stanley S. Stylli

**Affiliations:** 1 Department of Surgery, The University of Melbourne, The Royal Melbourne Hospital, Parkville, VIC 3050, Australia; sstylli@unimelb.edu.au or Stanley.stylli@mh.org.au; 2 Department of Neurosurgery, The Royal Melbourne Hospital, Parkville, VIC 3050, Australia

**Keywords:** glioblastoma, glioma, temozolomide, radiotherapy, immunotherapy, novel therapy, personalized treatment, drug repurposing

## Abstract

Glioblastoma (GBM) is the most common primary central nervous system tumor in adults. It is a highly invasive disease, making it difficult to achieve a complete surgical resection, resulting in poor prognosis with a median survival of 12–15 months after diagnosis, and less than 5% of patients survive more than 5 years. Surgical, instrument technology, diagnostic and radio/chemotherapeutic strategies have slowly evolved over time, but this has not translated into significant increases in patient survival. The current standard of care for GBM patients involving surgery, radiotherapy, and concomitant chemotherapy temozolomide (known as the Stupp protocol), has only provided a modest increase of 2.5 months in median survival, since the landmark publication in 2005. There has been considerable effort in recent years to increase our knowledge of the molecular landscape of GBM through advances in technology such as next-generation sequencing, which has led to the stratification of the disease into several genetic subtypes. Current treatments are far from satisfactory, and studies investigating acquired/inherent resistance to current therapies, restricted drug delivery, inter/intra-tumoral heterogeneity, drug repurposing and a tumor immune-evasive environment have been the focus of intense research over recent years. While the clinical advancement of GBM therapeutics has seen limited progression compared to other cancers, developments in novel treatment strategies that are being investigated are displaying encouraging signs for combating this disease. This aim of this editorial is to provide a brief overview of a select number of these novel therapeutic approaches.

## 1. Introduction

It is more than 90 years since Percival Bailey and Harvey Cushing published the first classification of brain tumors [1] and devised the term ‘glioblastoma multiforme’, even though gliomas had been previously documented [2]. Gliomas are the most common malignant tumor in adults and they account for approximately 80% of all brain-related malignancies [3]. The twenty-second (22nd) statistical report (2012–2016; 408,133 records) published by CBTRUS (Central Brain Tumor Registry of the United States) is the largest population based primary brain tumor/central nervous system (CNS) tumor registry in the United States [3]. The average annual age-adjusted incidence rate of malignant brain/other CNS tumors was 7.08 per 100,000 and the most commonly occurring malignant brain/other CNS tumor was GBM (14.6% of all tumors; 48.3% of all malignant tumors; 25,510 malignant tumors expected in 2019). GBM also accounted for the majority of all gliomas (57.3%) with an incidence rate of 3.22 per 100,000. The five-year relative survival rate following diagnosis of a malignant brain/other CNS tumor was 35.8%, but this was significantly lower for GBM at 6.8%. The incidence also increases with age, with a median of 65 years. Surgical resection alone provided a survival benefit of approximately 3–6 months, which increased to 12.1 months with the inclusion of radiotherapy treatment and a further slight increase to 14.6 months was observed with the addition of concomitant and adjuvant temozolomide [4].

The World Health Organization (WHO), classifies brain tumors using a grading system, with grade I being the least aggressive and the best prognosis, to grade IV being the most malignant with the worst prognosis [5]. GBM can present as a de novo primary tumor (approximately 90% of GBM patients), without histological/clinical evidence of a lower grade lesion, or as a secondary GBM arising from lower grade gliomas, such as a diffuse astrocytoma or anaplastic astrocytoma. Primary and secondary GBMs are histopathologically indistinguishable; however, secondary GBM patients are generally younger, present with a more favorable prognosis, and differ in their molecular signature [6]. In 2010, The Cancer Genome Atlas (TCGA), presented a multidimensional analysis of 216 GBM tumor samples with the aim of characterizing the GBM genomic landscape. Several major genomic alterations were identified. Epidermal Growth Factor Receptor (EGFR) amplification/mutations, Phosphatase and tensin homolog (PTEN) deletion/mutations and CDKN2Ap16^INK4a^ were most frequently observed in primary GBM, whereas the genomic alterations common to secondary GBM included isocitrate dehydrogenase 1/2 (IDH1/2) or Tumor protein 53 (TP53) mutations [6,7]. IDH1 was also identified as the most reliable diagnostic molecular marker of secondary GBM, as the mutation occurred more frequently in secondary GBM patients which correlated with an improved overall survival [6]. 

Large scale genomic studies such as the TCGA led to the identification of four GBM clinical subtypes—mesenchymal, classical, proneural, and neural, characterized by abnormalities in EGFR, IDH1, neurofibromin 1 (NF1), and platelet-derived growth factor receptor A (PDGFRA). Mesenchymal GBMs display an overexpression of mesenchymal and astrocytic markers, in addition to an NF1 deletion, and are seen in older patients with a poor prognosis. The classical subtype is associated with EGFR amplification, is highly proliferative and observed in older patients, also with a poor prognosis. Aggressive, higher-grade tumors are associated with these two subtypes. Proneural and neural subtype GBMs are generally seen in younger patients, present with IDH1, PDGFRA, PIK3C, TP53 alterations (proneural), or genes involved in nervous system development (neural) and are less aggressive tumors. Subsequently, a new classification was proposed by Verhaak [8], ultimately leading to a 2016 update of the WHO Classification of CNS tumors based on the integration of molecular parameters into diagnostic procedures previously based only on histopathological features [9]. This molecular-based approach is critical in determining the potential response to current treatment protocols that may influence patient prognosis and the design and implementation of appropriately targeted therapies.

## 2. Therapeutic Strategies for Glioblastoma

### 2.1. Targeted Therapies

With the advancement of next-generation sequencing and the comprehensive molecular mapping of GBM, several potential targets have been identified and various strategies are being evaluated as treatments for GBM. IDH mutations, which exist in high numbers in secondary GBM, involve both a loss and gain of enzyme function [10]. There is an abnormal accumulation of 2-hydroxyglutarate (2-HG), which is a driver of tumorigenesis [11,12]. Several IDH inhibitors are currently being evaluated in clinical trials, including AG-120 (mIDH1 inhibitor), AG881 (non-specific IDH inhibitor), FT-21-2 (mIDH1 inhibitor), and IDH305 (an IDH1(R132H inhibitor). EGFR inhibitors such as gefitinib, erlotinib, and afatinib have failed to show a survival benefit in GBM [13,14,15], even though they have been successful in other cancers. The activation of multiple receptor tyrosine kinase (RTK) pathways in GBM has also been proposed as a roadblock for single target-based strategies; therefore, efforts have been made to evaluate small molecule inhibitors with multiple targets such as Regorafenib. A phase II trial showed an increase in overall survival for recurrent GBM [16], while a current international phase I/II trial (GBM AGILE) is evaluating regorafenib with multiple treatment parameters for newly and recurrent GBM [17]. 

Depatuxizumab Mafodotin, also known as ABT-414, is an investigational anti-EGFR monoclonal antibody drug conjugate. ABT-414 targets the tumor cells by linking the anti-microtubule agent, monomethyl auristatin F, with an antibody directed against EGFR or mutant EGFRvIII. Participants within a phase I cohort who displayed EGFR amplification had a confirmed response, and this is currently being investigated in a phase II trial with ABT-414 and temozolomide in recurrent EGFR-amplified GBM. Monoclonal antibodies represent another class of targeted agents that have been used because of their high specificity and affinity to their targets. Bevacizumab, which binds to VEGF (vascular endothelial growth factor), inhibiting the growth of blood vessels, received accelerated FDA approval after encouraging phase I/II trials, but while phase III studies showed some extended progression-free survival, there was no observed overall survival benefit [18,19,20]. Cetuximab (EGFR monoclonal antibody), also failed to show survival benefits in phase II trials [21,22], identifying a potential weakness in the monoclonal antibody treatment strategy with incomplete tumor penetrance due to their size and restricted ability in crossing the blood brain barrier. 

### 2.2. Chemotherapy

Since the landmark study in 2005 by Stupp [23], TMZ has been the first-line treatment following surgery and radiotherapy. This randomized clinical study demonstrated a significant survival benefit with the addition of TMZ to radiotherapy (27.2% versus 10.9% survival at 2 years). However, not all GBM patients respond to this treatment known as the Stupp protocol, while others may eventually display innate or acquired chemoresistance, ultimately resulting in tumor recurrence [24]. A positive prognostic indicator for TMZ-based chemotherapy for newly diagnosed GBM was correlated with MGMT gene methylation [25]. 

The DIRECTOR trial, investigating alternative schedules of TMZ treatment, found no difference in outcome between their treatment protocols, but they also observed that MGMT promoter methylation was a prognostic marker in the TMZ treatment of recurrent GBM patients [26]. DNA alkylating agents, known as nitrosoureas including lomustine (CCNU), carmustine (BCNU), and nimustine (ACNU) have been used in the treatment of GBM, but they are generally avoided due to the presence of systemic side effects including suppression of bone marrow and severe kidney/liver toxicities. However, improvement in the survival of recurrent and newly diagnosed GBM patients has been recently observed with the placement of carmustine wafers in the resection cavity, reducing systemic side effects [27]. Nevertheless, it is anticipated that the clinical efficacy of nitrosourea-based treatment protocols will be more prominent in GBM patients with tumors displaying MGMT promoter methylation [28,29]. 

Since the development of new therapeutics is associated with high costs and slow progress to successful implementation in the clinic, drug repurposing has emerged as an attractive strategy, due to lower costs and a shortened time for transition to the clinic for a new indication. For example, a study trialing Metformin, which is utilized in the management for diabetes mellitus type 2, demonstrated that the progression-free survival of patients with GBM and metformin-treated diabetes was significantly increased [30]. Furthermore, a combined analysis of 1731 patients in the AVAglio, CENTRIC, and CORE trials did not demonstrate a significant improvement in overall survival with metformin, but there was a significant hazard ratio observed for progression-free survival in these patients at baseline [31]. Nonsteroidal anti-inflammatory drugs such as celecoxib have been investigated due to encouraging results in pre-clinical laboratory-based studies [32,33]. The inclusion of celecoxib as an adjuvant to therapeutics such as temozolomide, while showing good tolerability, was inconclusive in terms of providing a significant survival benefit [34]. Currently, the DIRECT phase II/III multicenter trial is examining the efficacy of disulfiram (potent inhibitor of aldehyde dehydrogenase) in a randomized controlled study with GBM patients, due for primary completion at the end of 2021 [35]. 

Historically, a single-target, single-drug strategy has been the focus of drug discovery, laboratory-based studies, and clinical treatment. However, due to the genetic heterogeneity of GBM tumors, a multitarget approach with the repurposing of several drugs as a pharmacological treatment protocol has been considered and is underway. This was initially known as the CUSP9 trial, but it has undergone several modifications and is now known as CUSPv3 [36,37]. The genomic profiling of GBM tumors, coupled with the bioinformatic match-up of molecular abnormalities with drug libraries and the corresponding known drug targets in designing a personalized drug cocktail is being evaluated [38]. Numerous chemotherapeutic agents are under investigation, and it is beyond this editorial to discuss and list all the completed and ongoing trials. This information is available through the website www.clinicaltrials.gov. 

### 2.3. Tumor Treating Fields (TTFields)

In 2011, a treatment technology known as tumor treating fields (TTFs), which utilizes intermediate frequency (200 KHz), low-intensity (1 V/cm) continuously delivered electric fields [39] to selectively target proliferating tumor cells by inhibiting mitosis was approved for the treatment of recurrent GBM by the FDA [40]. The first TTF device approved by the FDA, known as NovoTTF-100A (Optune^®^), manufactured by Novocure, is patient-operated, with the field generator being mounted on their shaved scalp. The results from the initial trials appear to be encouraging; when TTF was combined with TMZ chemotherapy, a significant increase in overall survival (20.9 months vs. 16 months) [41] compared to TMZ alone was observed, forming the foundation for further ongoing trials examining the efficacy of combining TTFields with chemotherapy in the treatment of GBM.

### 2.4. Laser Interstitial Therapy

Occasionally, GBM patients may not be candidates for surgical debulking of the tumor via an open craniotomy, and a relatively new technique known as ‘Laser Interstitial Thermal Therapy’ (LITT) is being trialed as a potential cytoreductive technique in destroying tumor cells via a localized elevated temperature [42,43,44]. It involves the insertion of an MRI-guided laser-tip probe into the tumor to deliver low-powered laser-induced thermotherapy. The initial studies have demonstrated that this therapy is safe [42], and an improved survival observed for patients with tumors where difficult surgical access may be achievable [45,46,47,48]. 

### 2.5. Radiotherapy

The current standard of care for GBM involves the combination of radiotherapy with chemotherapy [23]. Traditionally, whole brain radiation therapy was used. However due to the side effects of exposure of the normal brain to radiation, such as cognitive impairment, current practice utilizes focal radiotherapy treatment. The total radiotherapy dose of 60 Gy is normally delivered over 30 fractions of 2 Gy with adjuvant temozolomide [23], with the fractionated treatment allowing normal brain cells surrounding the tumor treatment area to recover between each treatment. Radiation dose escalation attempts have resulted in increased tissue damage and side effects, with no significant change in survival [49], hence there has been an effort in exploring other potential radiotherapy based strategies. Interstitial brachytherapy which requires the placement of radioactive isotopes (or seeds) into the surgical cavity is not an entirely new treatment, but due to continuing concerns such as radiation leakage into the surrounding brain, efforts into improving brachytherapy are underway, including the prolonged delivery of higher doses of radiation, use of alternative isotopes, and targeted delivery via the combination of isotopes with monoclonal antibodies. A treatment known as GammaTile, which involves inserting encapsulated radioactive cesium-131 seeds into the surgical cavity, was recently approved by the FDA for the treatment of GBM and has to date demonstrated feasibility and safety [50]. 

Proton Beam Therapy (PBT) has also been investigated as a therapeutic option for GBM, as the associated ‘Bragg Peak Effect’ reduces radiation exposure to the surrounding brain with the use of smaller treatment target volumes, providing for a lower risk of side effects such as neurocognitive decline. Dose escalation studies have been performed, with some observed toxicities [51,52], but it has also been shown to be a safe treatment option, resulting in a slight survival benefit for recurrent GBM [53]. Phase II trials are currently underway, evaluating the efficacy of PBT as a frontline treatment compared to standard dose radiotherapy with TMZ. The delivery of high dose radiation to the tumor can also be achieved via Gamma Knife Radiosurgery, which has been utilized for the treatment of recurrent GBM [54,55,56]. It has been observed that significant radiation-induced edema occurs in patients who receive high radiation doses; however, these adverse side effects were reduced, and patient survival prolonged when combined with bevacizumab [57,58].

### 2.6. Immunotherapies

Given the success that has been demonstrated with immunotherapeutic strategies in treating various cancers, there has been considerable effort into also translating this into a treatment for GBM patients. Traditionally, the brain is considered an immune-privileged organ due to the existence of the blood brain barrier (BBB) and the absence of a lymphatic drainage system. However, anti-tumor immune responses have been observed in brain tumors [59], which are proposed to be facilitated by the presence of a lymphatic system [60]. In general, immunotherapy has been more successful in treating tumors with a high mutational burden [61], but GBM has a low tumor mutational burden, while also displaying an immunosuppressive environment [62], and the added complication that chemotherapeutics can also promote an immunosuppressive effect [63]. Nevertheless, as immunotherapy involves harnessing the immune system to eradicate tumor cells, several different strategies have been explored with the goal to boost host immunity against GBM.

Immune checkpoint blockade has been utilized to achieve stimulation of the immune system with a significant effort focusing on blocking the binding of checkpoint receptors on immune cells such as Cytotoxic T-Lymphocyte Antigen 4 (CTLA-4) (early T-cell inhibition) and Programmed Cell Death protein 1 (PD-1) (late T-cell inhibition) to their corresponding ligands on tumor cells promoting a more effective T cell response against the tumor [64]. A number of checkpoint inhibitors that have been approved for use in several cancers have been trialed in the treatment of recurrent GBM, including nivolumab, pembrolizumab, durvalumab, atezolizumab [65]. The preliminary results have been less than inspiring; however, there are ongoing investigations into studying biomarkers that may identify which patients may respond to checkpoint blockade, the mutational load of the tumor as a predictor of response, administration of PD-1 antibodies prior to tumor resection to induce an early anti-tumor response, or analyze the effects of radiotherapy, which may be a synergistic facilitator of response to immunotherapy [66,67,68,69].

T-cell therapy involves the use of autologous T-cells, which are genetically engineered to express chimeric antigen receptor (CAR) constructs and have been FDA-approved for the treatment of hematologic malignancies. Several phase I trials have shown encouraging signs in terms of safety, feasibility and potential efficacy against relevant GBM surface antigens including IL13Ra2, HER2, EphA2 and EGFRVIII [70]. Even though the initial results have been promising, it is anticipated that, due to the high degree of heterogeneity exhibited by GBM tumors, T-cell therapy will be administered as a combination therapy, potentially with immune checkpoint blockade.

Vaccine-based strategies are also being investigated as a potential adoptive immunotherapy for GBM by stimulating an antigen-specific effector T cell response against tumor specific antigens (TSA) or tumor associated antigens (TAA). Several approaches have been utilized including cell-based protocols (patient-derived dendritic cell and autologous tumor cell vaccines) and non-cell based protocols (peptide and heat shock protein vaccines). Engineered peptide sequences that provide a targeted immunity against tumor associated antigens bound to major histocompatibility complexes form the basis of peptide vaccines. An example of two peptide vaccines are rindopepimut (EGFRvIII) [71,72,73] and SurVaxM (Survivin) [74,75]. While rindopepimut showed impressive responses in the early-phase studies [76], a survival benefit was not observed in the phase III evaluation [77]. However, a separate phase II study combining rindopepimut with temozolomide improved progression-free and overall survival for GBM patients [72], as well as the demonstration of encouraging results in a phase II study combining rindopepimut with bevacizumab in the treatment of recurrent GBM patients [78]. A phase II study evaluating SurVaxM has displayed improvements in progression-free and overall survival [74]. 

Heat shock proteins have also been utilized to deliver a variety of tumor antigens and are designed to create an anti-tumor inflammatory response. HSPPC-96 is one such vaccine, which has undergone a phase II, multicenter clinical trial for recurrent GBM [79]. Autologous tumor cell based vaccines use cytotoxic T lymphocytes that are induced with patient-derived tumor cells, which then subsequently elicit an immune response, once they are reintroduced back into the patient [80,81]. Dendritic cell vaccines rely on patient-derived dendritic cells that are exposed to purified tumor-specific antigens or tumor cell extracts derived from the tumor before being reintroduced to the patient, subsequently activating CD8+ and CD4+ T cells. A phase I trial with an autologous dendritic cell vaccine has demonstrated a correlation between the expression level of tumor-associated antigens on the glioma cells and prolonged overall/progression free patient survival [82]. Viral-based therapy that involves delivery of the gene of interest via viral vectors is also being investigated as a form of immunotherapy for treating GBM. Oncolytic viruses can selectively replicate in tumor cells, eliciting cytotoxic effects, ultimately providing an immunostimulatory effect. DNX-2401 is a replication-competent adenovirus that uses tumor-specific integrins to produce oncolytic effects [83,84], whereas PVSRIPO (attenuated polio-rhinovirus chimera) recognizes CD155 (poliovirus receptor), which is widely expressed in tumor cells [85,86].

## 3. Conclusions

The treatment of GBM continues to be a complex and difficult challenge. Previous attempts to find a cure have only resulted in a slight improvement in survival over the last 50 years, as the current 5-year survival rate remains low at <10% [25]. As there are limitations on the number of times the current therapeutic approach of surgery, radiotherapy, and chemotherapy can be utilized, the ideal novel therapeutic agent or treatment protocol, as part of a multimodal strategy, must function to eliminate any residual tumor. Ultimately, this may be achieved by the synergistic effects of combining a number of the current therapeutic strategies briefly outlined in this editorial, including a targeted therapy, immunotherapy, chemotherapy, or radiotherapy, as treatment resistance can potentially develop to a single therapy. The development of new and novel therapies has been aided by the considerable efforts to decipher the genomic landscape of GBM with the evolution of next generation sequencing, leading to modifications in tumor classification and the ‘molecular’ clinical management of some GBM patients. 

Over time, the therapeutic options available will increase with additional targetable and actionable combinations of genomic mutations and alterations being uncovered, as only a small fraction to date have been demonstrated to have clinical implementation. Importantly, as tumor heterogeneity and patient-to-patient variability contributing to the growth of GBM and response to treatment is driven by the genomics of each tumor, a personalized treatment approach through the stratification of patients into molecular subgroups will be critical in their allocation to the most appropriate novel treatment strategy that will be available in the future management of GBM. The continued collaboration between researchers and clinicians, coupled with advancements in technology, both scientifically and clinically, provides for an optimistic future that new and effective treatments will be developed for GBM patients. 

## References

[1] Bailey P.C.H. (1926). A Classification of the Tumors of the Glioma Group on a Histogenetic Basis with a Correlated Study of Prognosis.

[2] Bailey P., Cushing H. (1925). Microchemical Color Reactions as an Aid to the Identification and Classification of Brain Tumors. Proc. Natl. Acad. Sci. USA.

[3] Ostrom Q.T., Cioffi G., Gittleman H., Patil N., Waite K., Kruchko C., Barnholtz–Sloan J.S. (2019). CBTRUS Statistical Report: Primary Brain and Other Central Nervous System Tumors Diagnosed in the United States in 2012–2016. Neuro Oncol..

[4] Agnihotri S., Burrell K.E., Wolf A., Jalali S., Hawkins C., Rutka J.T., Zadeh G. (2013). Glioblastoma, a brief review of history, molecular genetics, animal models and novel therapeutic strategies. Arch. Immunol. Ther. Exp. (Warsz).

[5] Louis D.N., Ohgaki H., Wiestler O.D., Cavenee W.K., Burger P.C., Jouvet A., Scheithauer B.W., Kleihues P. (2007). The 2007 WHO classification of tumours of the central nervous system. Acta Neuropathol..

[6] Ohgaki H., Kleihues P. (2013). The definition of primary and secondary glioblastoma. Clin. Cancer Res..

[7] Jiao Y., Killela P.J., Reitman Z.J., Rasheed A.B., Heaphy C.M., de Wilde R.F., Rodriguez F.J., Rosemberg S., Oba–Shinjo S.M., Nagahashi Marie S.K. (2012). Frequent ATRX, CIC, FUBP1 and IDH1 mutations refine the classification of malignant gliomas. Oncotarget.

[8] Verhaak R.G., Hoadley K.A., Purdom E., Wang V., Qi Y., Wilkerson M.D., Miller C.R., Ding L., Golub T., Mesirov J.P. (2010). Integrated genomic analysis identifies clinically relevant subtypes of glioblastoma characterized by abnormalities in PDGFRA, IDH1, EGFR, and NF1. Cancer Cell.

[9] Louis D.N., Perry A., Reifenberger G., von Deimling A., Figarella-Branger D., Cavenee W.K., Ohgaki H., Wiestler O.D., Kleihues P., Ellison D.W. (2016). The 2016 World Health Organization Classification of Tumors of the Central Nervous System: A summary. Acta Neuropathol..

[10] Cohen A.L., Holmen S.L., Colman H. (2013). IDH1 and IDH2 mutations in gliomas. Curr. Neurol. Neurosci. Rep..

[11] Killock D. (2016). CNS cancer: Breaking boundaries—IDH mutations in glioma. Nat. Rev. Clin. Oncol..

[12] Kim W., Liau L.M. (2012). IDH mutations in human glioma. Neurosurg. Clin. N. Am..

[13] Sepulveda–Sanchez J.M., Vaz M.A., Balana C., Gil–Gil M., Reynes G., Gallego O., Martinez–Garcia M., Vicente E., Quindos M., Luque R. (2017). Phase II trial of dacomitinib, a pan–human EGFR tyrosine kinase inhibitor, in recurrent glioblastoma patients with EGFR amplification. Neuro Oncol..

[14] Kreisl T.N., Lassman A.B., Mischel P.S., Rosen N., Scher H.I., Teruya–Feldstein J., Shaffer D., Lis E., Abrey L.E. (2009). A pilot study of everolimus and gefitinib in the treatment of recurrent glioblastoma (GBM). J. Neurooncol..

[15] Reardon D.A., Nabors L.B., Mason W.P., Perry J.R., Shapiro W., Kavan P., Mathieu D., Phuphanich S., Cseh A., Fu Y. (2015). Phase I/randomized phase II study of afatinib, an irreversible ErbB family blocker, with or without protracted temozolomide in adults with recurrent glioblastoma. Neuro Oncol..

[16] Lombardi G., De Salvo G.L., Brandes A.A., Eoli M., Ruda R., Faedi M., Lolli I., Pace A., Daniele B., Pasqualetti F. (2019). Regorafenib compared with lomustine in patients with relapsed glioblastoma (REGOMA): A multicentre, open–label, randomised, controlled, phase 2 trial. Lancet Oncol..

[17] Alexander B.M., Ba S., Berger M.S., Berry D.A., Cavenee W.K., Chang S.M., Cloughesy T.F., Jiang T., Khasraw M., Li W. (2018). Adaptive Global Innovative Learning Environment for Glioblastoma: GBM AGILE. Clin. Cancer Res..

[18] Gilbert M.R., Sulman E.P., Mehta M.P. (2014). Bevacizumab for newly diagnosed glioblastoma. N. Engl. J. Med..

[19] Hamza M.A., Mandel J.J., Conrad C.A., Gilbert M.R., Yung W.K., Puduvalli V.K., DeGroot J.F. (2014). Survival outcome of early versus delayed bevacizumab treatment in patients with recurrent glioblastoma. J. Neurooncol..

[20] Gilbert M.R., Dignam J.J., Armstrong T.S., Wefel J.S., Blumenthal D.T., Vogelbaum M.A., Colman H., Chakravarti A., Pugh S., Won M. (2014). A randomized trial of bevacizumab for newly diagnosed glioblastoma. N. Engl. J. Med..

[21] Neyns B., Sadones J., Joosens E., Bouttens F., Verbeke L., Baurain J.F., D’Hondt L., Strauven T., Chaskis C., In’t Veld P. (2009). Stratified phase II trial of cetuximab in patients with recurrent high–grade glioma. Ann. Oncol..

[22] Hasselbalch B., Lassen U., Hansen S., Holmberg M., Sorensen M., Kosteljanetz M., Broholm H., Stockhausen M.T., Poulsen H.S. (2010). Cetuximab, bevacizumab, and irinotecan for patients with primary glioblastoma and progression after radiation therapy and temozolomide: A phase II trial. Neuro Oncol..

[23] Stupp R., Mason W.P., van den Bent M.J., Weller M., Fisher B., Taphoorn M.J., Belanger K., Brandes A.A., Marosi C., Bogdahn U. (2005). Radiotherapy plus concomitant and adjuvant temozolomide for glioblastoma. N. Engl. J. Med..

[24] Lee S.Y. (2016). Temozolomide resistance in glioblastoma multiforme. Genes Dis..

[25] Stupp R., Hegi M.E., Mason W.P., van den Bent M.J., Taphoorn M.J., Janzer R.C., Ludwin S.K., Allgeier A., Fisher B., Belanger K. (2009). Effects of radiotherapy with concomitant and adjuvant temozolomide versus radiotherapy alone on survival in glioblastoma in a randomised phase III study: 5–year analysis of the EORTC–NCIC trial. Lancet Oncol..

[26] Weller M., Tabatabai G., Kastner B., Felsberg J., Steinbach J.P., Wick A., Schnell O., Hau P., Herrlinger U., Sabel M.C. (2015). MGMT Promoter Methylation Is a Strong Prognostic Biomarker for Benefit from Dose–Intensified Temozolomide Rechallenge in Progressive Glioblastoma: The DIRECTOR Trial. Clin. Cancer Res..

[27] Chowdhary S.A., Ryken T., Newton H.B. (2015). Survival outcomes and safety of carmustine wafers in the treatment of high–grade gliomas: A meta–analysis. J. Neurooncol..

[28] Taal W., van der Rijt C.C., Dinjens W.N., Sillevis Smitt P.A., Wertenbroek A.A., Bromberg J.E., van Heuvel I., Kros J.M., van den Bent M.J. (2015). Treatment of large low–grade oligodendroglial tumors with upfront procarbazine, lomustine, and vincristine chemotherapy with long follow–up: A retrospective cohort study with growth kinetics. J. Neurooncol..

[29] Taal W., Oosterkamp H.M., Walenkamp A.M., Dubbink H.J., Beerepoot L.V., Hanse M.C., Buter J., Honkoop A.H., Boerman D., de Vos F.Y. (2014). Single–agent bevacizumab or lomustine versus a combination of bevacizumab plus lomustine in patients with recurrent glioblastoma (BELOB trial): A randomised controlled phase 2 trial. Lancet Oncol..

[30] Adeberg S., Bernhardt D., Ben Harrabi S., Bostel T., Mohr A., Koelsche C., Diehl C., Rieken S., Debus J. (2015). Metformin influences progression in diabetic glioblastoma patients. Strahlenther. Onkol..

[31] Seliger C., Genbrugge E., Gorlia T., Chinot O., Stupp R., Nabors B., Weller M., Hau P., Group E.B.T. (2020). Use of metformin and outcome of patients with newly diagnosed glioblastoma: Pooled analysis. Int. J. Cancer.

[32] Sareddy G.R., Kesanakurti D., Kirti P.B., Babu P.P. (2013). Nonsteroidal anti–inflammatory drugs diclofenac and celecoxib attenuates Wnt/beta–catenin/Tcf signaling pathway in human glioblastoma cells. Neurochem. Res..

[33] Chirasani S.R., Leukel P., Gottfried E., Hochrein J., Stadler K., Neumann B., Oefner P.J., Gronwald W., Bogdahn U., Hau P. (2013). Diclofenac inhibits lactate formation and efficiently counteracts local immune suppression in a murine glioma model. Int. J. Cancer.

[34] Stockhammer F., Misch M., Koch A., Czabanka M., Plotkin M., Blechschmidt C., Tuettenberg J., Vajkoczy P. (2010). Continuous low–dose temozolomide and celecoxib in recurrent glioblastoma. J. Neurooncol..

[35] Jakola A.S., Werlenius K., Mudaisi M., Hylin S., Kinhult S., Bartek J., Salvesen O., Carlsen S.M., Strandeus M., Lindskog M. (2018). Disulfiram repurposing combined with nutritional copper supplement as add–on to chemotherapy in recurrent glioblastoma (DIRECT): Study protocol for a randomized controlled trial. F1000Res.

[36] Kast R.E., Karpel–Massler G., Halatsch M.E. (2014). CUSP9* treatment protocol for recurrent glioblastoma: Aprepitant, artesunate, auranofin, captopril, celecoxib, disulfiram, itraconazole, ritonavir, sertraline augmenting continuous low dose temozolomide. Oncotarget.

[37] Skaga E., Skaga I.O., Grieg Z., Sandberg C.J., Langmoen I.A., Vik–Mo E.O. (2019). The efficacy of a coordinated pharmacological blockade in glioblastoma stem cells with nine repurposed drugs using the CUSP9 strategy. J. Cancer Res. Clin. Oncol..

[38] Byron S.A., Tran N.L., Halperin R.F., Phillips J.J., Kuhn J.G., de Groot J.F., Colman H., Ligon K.L., Wen P.Y., Cloughesy T.F. (2018). Prospective Feasibility Trial for Genomics–Informed Treatment in Recurrent and Progressive Glioblastoma. Clin. Cancer Res..

[39] Wenger C., Salvador R., Basser P.J., Miranda P.C. (2015). The electric field distribution in the brain during TTFields therapy and its dependence on tissue dielectric properties and anatomy: A computational study. Phys. Med. Biol..

[40] Davies A.M., Weinberg U., Palti Y. (2013). Tumor treating fields: A new frontier in cancer therapy. Ann. N. Y. Acad. Sci..

[41] Stupp R., Taillibert S., Kanner A., Read W., Steinberg D., Lhermitte B., Toms S., Idbaih A., Ahluwalia M.S., Fink K. (2017). Effect of Tumor–Treating Fields Plus Maintenance Temozolomide vs. Maintenance Temozolomide Alone on Survival in Patients With Glioblastoma: A Randomized Clinical Trial. JAMA.

[42] Kamath A.A., Friedman D.D., Akbari S.H.A., Kim A.H., Tao Y., Luo J., Leuthardt E.C. (2019). Glioblastoma Treated with Magnetic Resonance Imaging–Guided Laser Interstitial Thermal Therapy: Safety, Efficacy, and Outcomes. Neurosurgery.

[43] Kim A.H., Tatter S., Rao G., Prabhu S., Chen C., Fecci P., Chiang V., Smith K., Williams B.J., Mohammadi A.M. (2020). Laser Ablation of Abnormal Neurological Tissue Using Robotic NeuroBlate System (LAANTERN): 12–Month Outcomes and Quality of Life After Brain Tumor Ablation. Neurosurgery.

[44] Sloan A.E., Ahluwalia M.S., Valerio–Pascua J., Manjila S., Torchia M.G., Jones S.E., Sunshine J.L., Phillips M., Griswold M.A., Clampitt M. (2013). Results of the NeuroBlate System first–in–humans Phase I clinical trial for recurrent glioblastoma: Clinical article. J. Neurosurg..

[45] Thomas J.G., Rao G., Kew Y., Prabhu S.S. (2016). Laser interstitial thermal therapy for newly diagnosed and recurrent glioblastoma. Neurosurg. Focus.

[46] Rahmathulla G., Recinos P.F., Kamian K., Mohammadi A.M., Ahluwalia M.S., Barnett G.H. (2014). MRI–guided laser interstitial thermal therapy in neuro–oncology: A review of its current clinical applications. Oncology.

[47] Mohammadi A.M., Hawasli A.H., Rodriguez A., Schroeder J.L., Laxton A.W., Elson P., Tatter S.B., Barnett G.H., Leuthardt E.C. (2014). The role of laser interstitial thermal therapy in enhancing progression–free survival of difficult–to–access high–grade gliomas: A multicenter study. Cancer Med..

[48] Mohammadi A.M., Schroeder J.L. (2014). Laser interstitial thermal therapy in treatment of brain tumors—The NeuroBlate System. Expert Rev. Med. Devices.

[49] Barani I.J., Larson D.A. (2015). Radiation therapy of glioblastoma. Cancer Treat. Res..

[50] Gessler D.J., Ferreira C., Dusenbery K., Chen C.C. (2020). GammaTile((R)): Surgically targeted radiation therapy for glioblastomas. Future Oncol..

[51] Mizumoto M., Yamamoto T., Ishikawa E., Matsuda M., Takano S., Ishikawa H., Okumura T., Sakurai H., Matsumura A., Tsuboi K. (2016). Proton beam therapy with concurrent chemotherapy for glioblastoma multiforme: Comparison of nimustine hydrochloride and temozolomide. J. Neurooncol..

[52] Mizumoto M., Yamamoto T., Takano S., Ishikawa E., Matsumura A., Ishikawa H., Okumura T., Sakurai H., Miyatake S., Tsuboi K. (2015). Long–term survival after treatment of glioblastoma multiforme with hyperfractionated concomitant boost proton beam therapy. Pract. Radiat Oncol..

[53] Scartoni D., Amelio D., Palumbo P., Giacomelli I., Amichetti M. (2020). Proton therapy re–irradiation preserves health–related quality of life in large recurrent glioblastoma. J. Cancer Res. Clin. Oncol..

[54] Larson E.W., Peterson H.E., Lamoreaux W.T., MacKay A.R., Fairbanks R.K., Call J.A., Carlson J.D., Ling B.C., Demakas J.J., Cooke B.S. (2014). Clinical outcomes following salvage Gamma Knife radiosurgery for recurrent glioblastoma. World J. Clin. Oncol..

[55] Guseynova K., Liscak R., Simonova G., Novotny J. (2018). Gamma knife radiosurgery for local recurrence of glioblastoma. Neuro Endocrinol. Lett..

[56] Brehmer S., Grimm M.A., Forster A., Seiz–Rosenhagen M., Welzel G., Stieler F., Wenz F., Groden C., Mai S., Hanggi D. (2019). Study Protocol: Early Stereotactic Gamma Knife Radiosurgery to Residual Tumor After Surgery of Newly Diagnosed Glioblastoma (Gamma–GBM). Neurosurgery.

[57] Koga T., Saito N. (2012). Efficacy and limitations of stereotactic radiosurgery in the treatment of glioblastoma. Neurol. Med. Chir. (Tokyo).

[58] Koga T., Maruyama K., Tanaka M., Ino Y., Saito N., Nakagawa K., Shibahara J., Todo T. (2012). Extended field stereotactic radiosurgery for recurrent glioblastoma. Cancer.

[59] D’Alessio A., Proietti G., Sica G., Scicchitano B.M. (2019). Pathological and Molecular Features of Glioblastoma and Its Peritumoral Tissue. Cancers (Basel).

[60] Song E., Mao T., Dong H., Boisserand L.S.B., Antila S., Bosenberg M., Alitalo K., Thomas J.L., Iwasaki A. (2020). VEGF–C–driven lymphatic drainage enables immunosurveillance of brain tumours. Nature.

[61] Riviere P., Goodman A.M., Okamura R., Barkauskas D.A., Whitchurch T.J., Lee S., Khalid N., Collier R., Marebiona M., Frampton G.M. (2020). High Tumor Mutational Burden Correlates with Longer Survival in Immunotherapy–Naive Patients with Diverse Cancers. Mol. Cancer Ther..

[62] Liu E.K., Sulman E.P., Wen P.Y., Kurz S.C. (2020). Novel Therapies for Glioblastoma. Curr. Neurol. Neurosci. Rep..

[63] Sengupta S., Marrinan J., Frishman C., Sampath P. (2012). Impact of temozolomide on immune response during malignant glioma chemotherapy. Clin. Dev. Immunol..

[64] Maxwell R., Jackson C.M., Lim M. (2017). Clinical Trials Investigating Immune Checkpoint Blockade in Glioblastoma. Curr. Treat. Options Oncol..

[65] Romani M., Pistillo M.P., Carosio R., Morabito A., Banelli B. (2018). Immune Checkpoints and Innovative Therapies in Glioblastoma. Front. Oncol..

[66] Rajani K.R., Carlstrom L.P., Parney I.F., Johnson A.J., Warrington A.E., Burns T.C. (2018). Harnessing Radiation Biology to Augment Immunotherapy for Glioblastoma. Front. Oncol..

[67] Le D.T., Durham J.N., Smith K.N., Wang H., Bartlett B.R., Aulakh L.K., Lu S., Kemberling H., Wilt C., Luber B.S. (2017). Mismatch repair deficiency predicts response of solid tumors to PD–1 blockade. Science.

[68] Bouffet E., Larouche V., Campbell B.B., Merico D., de Borja R., Aronson M., Durno C., Krueger J., Cabric V., Ramaswamy V. (2016). Immune Checkpoint Inhibition for Hypermutant Glioblastoma Multiforme Resulting From Germline Biallelic Mismatch Repair Deficiency. J. Clin. Oncol..

[69] Cloughesy T.F., Mochizuki A.Y., Orpilla J.R., Hugo W., Lee A.H., Davidson T.B., Wang A.C., Ellingson B.M., Rytlewski J.A., Sanders C.M. (2019). Neoadjuvant anti–PD–1 immunotherapy promotes a survival benefit with intratumoral and systemic immune responses in recurrent glioblastoma. Nat. Med..

[70] Bagley S.J., Desai A.S., Linette G.P., June C.H., O’Rourke D.M. (2018). CAR T–cell therapy for glioblastoma: Recent clinical advances and future challenges. Neuro Oncol..

[71] Zussman B.M., Engh J.A. (2015). Outcomes of the ACT III Study: Rindopepimut (CDX–110) Therapy for Glioblastoma. Neurosurgery.

[72] Schuster J., Lai R.K., Recht L.D., Reardon D.A., Paleologos N.A., Groves M.D., Mrugala M.M., Jensen R., Baehring J.M., Sloan A. (2015). A phase II, multicenter trial of rindopepimut (CDX–110) in newly diagnosed glioblastoma: The ACT III study. Neuro Oncol..

[73] Swartz A.M., Li Q.J., Sampson J.H. (2014). Rindopepimut: A promising immunotherapeutic for the treatment of glioblastoma multiforme. Immunotherapy.

[74] Fenstermaker R.A., Ciesielski M.J., Qiu J., Yang N., Frank C.L., Lee K.P., Mechtler L.R., Belal A., Ahluwalia M.S., Hutson A.D. (2016). Clinical study of a survivin long peptide vaccine (SurVaxM) in patients with recurrent malignant glioma. Cancer Immunol. Immunother..

[75] Fenstermaker R.A., Ciesielski M.J. (2014). Challenges in the development of a survivin vaccine (SurVaxM) for malignant glioma. Expert Rev. Vaccines.

[76] Del Vecchio C.A., Wong A.J. (2010). Rindopepimut, a 14–mer injectable peptide vaccine against EGFRvIII for the potential treatment of glioblastoma multiforme. Curr. Opin. Mol. Ther..

[77] Weller M., Butowski N., Tran D.D., Recht L.D., Lim M., Hirte H., Ashby L., Mechtler L., Goldlust S.A., Iwamoto F. (2017). Rindopepimut with temozolomide for patients with newly diagnosed, EGFRvIII–expressing glioblastoma (ACT IV): A randomised, double–blind, international phase 3 trial. Lancet Oncol..

[78] Reardon D.A., Desjardins A., Vredenburgh J.J., O’Rourke D.M., Tran D.D., Fink K.L., Nabors L.B., Li G., Bota D.A., Lukas R.V. (2020). Rindopepimut with Bevacizumab for Patients with Relapsed EGFRvIII–Expressing Glioblastoma (ReACT): Results of a Double–Blind Randomized Phase II Trial. Clin. Cancer Res..

[79] Schuster J.L.R., Recht L.D., Reardon D.A., Paleologos N.A., Groves M.D. (2014). Heat–shock protein peptide complex–96 vaccination for recurrent glioblastoma: A phase II, single–arm trial. Neuro Oncol..

[80] Chamberlain M.C. (2014). Is there a role for vaccine–based therapy in recurrent glioblastoma?. Neuro Oncol..

[81] Reardon D.A., Wucherpfennig K.W., Freeman G., Wu C.J., Chiocca E.A., Wen P.Y., Curry W.T., Mitchell D.A., Fecci P.E., Sampson J.H. (2013). An update on vaccine therapy and other immunotherapeutic approaches for glioblastoma. Expert Rev. Vaccines.

[82] Phuphanich S., Wheeler C.J., Rudnick J.D., Mazer M., Wang H., Nuno M.A., Richardson J.E., Fan X., Ji J., Chu R.M. (2013). Phase I trial of a multi–epitope–pulsed dendritic cell vaccine for patients with newly diagnosed glioblastoma. Cancer Immunol. Immunother..

[83] Lang F.F., Conrad C., Gomez–Manzano C., Yung W.K.A., Sawaya R., Weinberg J.S., Prabhu S.S., Rao G., Fuller G.N., Aldape K.D. (2018). Phase I Study of DNX–2401 (Delta–24–RGD) Oncolytic Adenovirus: Replication and Immunotherapeutic Effects in Recurrent Malignant Glioma. J. Clin. Oncol..

[84] Philbrick B., Adamson D.C. (2019). DNX–2401: An investigational drug for the treatment of recurrent glioblastoma. Expert Opin. Investig. Drugs.

[85] Walton R.W., Brown M.C., Sacco M.T., Gromeier M. (2018). Engineered Oncolytic Poliovirus PVSRIPO Subverts MDA5–Dependent Innate Immune Responses in Cancer Cells. J. Virol..

[86] Desjardins A., Gromeier M., Herndon J.E., Beaubier N., Bolognesi D.P., Friedman A.H., Friedman H.S., McSherry F., Muscat A.M., Nair S. (2018). Recurrent Glioblastoma Treated with Recombinant Poliovirus. N. Engl. J. Med..

